# First report of *Acinetobacter* spp. and *Proteus mirabilis* isolated from aborted ovine fetuses

**DOI:** 10.29374/2527-2179.bjvm007225

**Published:** 2026-03-20

**Authors:** Huber Rizzo, Urias Fagner Santos Nascimento, Márcio Douglas Leal da Silveira, Emmylly Victória Gomes de Lima, Lorena Gabriela Rocha Ribeiro, Erika Fernanda Torres Samico-Fernandes, Valdemiro Amaro da Silva

**Affiliations:** 1 Departamento de Medicina Veterinária, Universidade Federal Rural de Pernambuco, Recife, PE, Brazil; 2 Faculdade Pio Décimo, Aracaju, SE, Brazil; 3 Programa de Residência em Medicina Veterinária, Universidade Federal Rural de Pernambuco, Recife, PE, Brazil; 4 Departamento de Medicina Veterinária, Universidade Federal de Sergipe, São Cristóvão, SE, Brazil

**Keywords:** hepatic abscess, microbiology, opportunistic bacteria, reproductive losses, sheep farming, abortamento, abscesso hepático, microbiologia, ovinocultura, perdas reprodutivas

## Abstract

This report describes two cases involving the isolation of *Acinetobacter spp.* and *Proteus mirabilis* from aborted ovine fetuses in Northeastern Brazil, representing the first documented occurrence of this association worldwide. In case 1, *Acinetobacter spp.* was isolated from a hepatic abscess in a White Dorper fetus, accompanied by placental lesions. In case 2, *A. baumannii* and *P. mirabilis* were isolated from the brain and lungs of three Santa Inês fetuses, with pathological findings consistent with acute septicemia. In both cases, bacteria were identified through culture and biochemical testing, without molecular confirmation, and no laboratory testing was conducted to exclude classical abortifacients (*Brucella* spp., *Campylobacter* spp., *Coxiella burnetii*, *Chlamydia abortus*, *Listeria* spp., *Salmonella* spp., *Toxoplasma gondii*, and *Neospora caninum*), representing a study limitation. Nevertheless, the results highlight the potential opportunistic role of these microorganisms, the detection of antimicrobial resistance profiles, and the importance of including them in the investigation of reproductive losses, particularly in settings with limited diagnostic resources. These findings reinforce the need for integrated surveillance in animal and public health under the “One Health” approach.

## Resumo

Este relato descreve dois casos envolvendo o isolamento de *Acinetobacter* spp. e *Proteus mirabilis* de fetos ovinos abortados no Nordeste do Brasil, representando a primeira ocorrência documentada dessa associação em nível mundial. No caso 1, *Acinetobacter* spp. foi isolado de um abscesso hepático em um feto White Dorper, acompanhado de lesões placentárias. No caso 2, *A. baumannii* e *P. mirabilis* foram isolados do cérebro e dos pulmões de três fetos Santa Inês, com achados patológicos compatíveis com septicemia aguda. Em ambos os casos, as bactérias foram identificadas por meio de cultura e testes bioquímicos, sem confirmação molecular, e não foram realizados exames laboratoriais para excluir abortifacientes clássicos (*Brucella* spp., *Campylobacter* spp., *Coxiella burnetii*, *Chlamydia abortus*, *Listeria* spp., *Salmonella* spp., *Toxoplasma gondii* e *Neospora caninum*), o que representa uma limitação do estudo. Ainda assim, os resultados destacam o potencial papel oportunista desses microrganismos, a detecção de perfis de resistência antimicrobiana e a importância de incluí-los na investigação de perdas reprodutivas, particularmente em cenários com recursos diagnósticos limitados. Esses achados reforçam a necessidade de vigilância integrada em saúde animal e pública sob a abordagem “One Health”.

## Introduction

*Acinetobacter* comprises Gram-negative, non-sporulating, and non-motile bacilli, which are widely distributed in the environment and recognized as emerging opportunistic pathogens in both human and veterinary medicine, mainly due to their ability to develop resistance to multiple antimicrobial classes (Howard et al., 2012; Ahuatzin-Flores et al., 2024). In ruminants, *Acinetobacter* spp. have been found in various tissues and fluids, including respiratory secretions, milk, and uterine contents (Diker et al., 1986; Garcia et al., 2013; Chen et al., 2024), generally associated with immunosuppression or pre-existing lesions. Although *Acinetobacter* spp. are detected in abortion cases in cattle, horses, and buffaloes (*A. lwoffii*, *A. calcoaceticus*, and *A. johnsonii*), its role as a primary causative agent remains poorly understood (Das & Paranjape, 1986; Arserim et al., 2020; Akter et al., 2021; Isogami et al., 2023; Wolf-Jäckel et al., 2020).

*Proteus mirabilis*, a member of the Enterobacteriaceae family, is another environmental and opportunistic microorganism well recognized for its role in urinary tract, cutaneous, and systemic infections in humans and animals (Chakkour et al., 2024). In small ruminants, sporadic reports have documented its involvement in mastitis (Lafta, 2020; Ali et al., 2021), pericarditis (Najd Ghahremani et al., 2023), and cutaneous infections (Abdollahi et al., 2022), generally in traumatic or immunosuppressive contexts. However, its contribution in abortion cases is rare and poorly investigated.

Research on reproductive losses in sheep traditionally focuses on classical abortifacients, such as *Brucella* spp., *Campylobacter* spp., *Coxiella burnetii*, *Chlamydia abortus*, *Listeria* spp., *Salmonella* spp., *Toxoplasma gondii*, and *Neospora caninum* (Concha-Bermejillo & Romano, 2021; Dorsch et al., 2022). However, in many settings, particularly in small- to medium-scale flocks in Brazil, the limited infrastructure and resources hinder the performance of specific laboratory tests, thereby restricting etiological diagnosis.

To the best of our knowledge, this study is the first to describe the isolation of *Acinetobacter* spp. and *P. mirabilis* from aborted ovine fetuses and the microbiological and histopathological characteristics of lesions. Although no laboratory tests were performed to exclude classical abortifacients and molecular confirmation of the isolates, the findings are relevant to broadening the etiological spectrum considered in abortion investigations, highlighting the potential opportunistic role of these microorganisms, and reinforcing the importance of integrated animal and public health surveillance under the “One Health” approach.

## Case report

**Figure 1:**
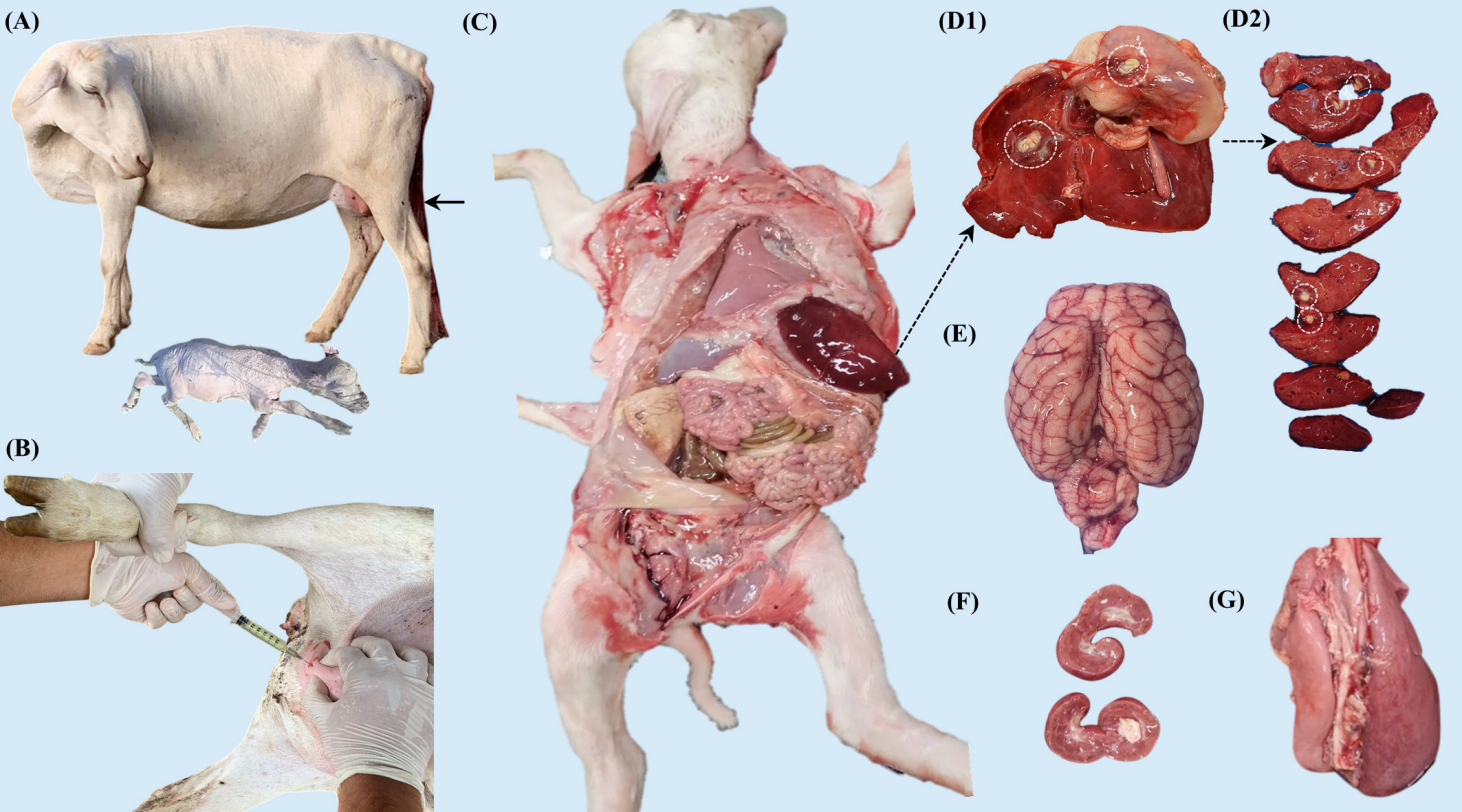
Macroscopic findings at a necropsy of an ovine abortion case with *Acinetobacter* spp. isolated from a hepatic abscess. (A) A ewe with partially exteriorized placenta protruding from the vulva (arrow) and an aborted lamb found in the pasture. (B) Aspiration of a mammary gland nodule, with the animal in the lateral recumbent position for exudate collection. (C) General view of the thoracic and abdominal cavities of the fetus, showing mild hydrothorax, ascites, and congested liver. (D1) Liver with multiple firm, whitish, well-demarcated abscesses (dotted circles) distributed multifocally, and (D2) cross-section showing yellowish-white purulent content. (E) Brain with diffuse hyperemia and bilaterally and symmetrically engorged meningeal vessels. (F) Kidneys with preserved architecture and coloration, presenting a normal appearance. (G) Lungs with homogeneous pink coloration without visible areas of consolidation and evidence of physiological atelectasis.

**Case 1:** In October 2023, *Acinetobacter* spp. was isolated from samples obtained from an aborted ovine fetus belonging to a flock of 30 animals raised under a semi-extensive management system in the municipality of Paulista, Pernambuco, Brazil (7°56′27′′ S, 34°52′22′′ W). During the morning inspection, a three-year-old White Dorper ewe, in her second lambing, was found with a placenta protruding from the vulva and a freshly aborted, late-gestation fetuses on the ground (Figure 1A). The ewe had a body condition score of 3 (scale 1–5), mild anorexia, and clinical signs of mastitis with mammary abscesses.

The fetus and placenta were collected, and under aseptic conditions, purulent exudate was obtained by puncturing a mammary abscess using a 25 × 0.70 mm needle attached to a 5-mL syringe, yielding thick, yellowish purulent material (Figure 1B). The samples were refrigerated in an aseptic, insulated container and sent to the pathology and infectious diseases laboratories at Federal Rural University of Pernambuco (UFRPE), Recife, Pernambuco, Brazil, for necropsy, histopathology, and microbiological analyses.

At necropsy, mild hydrothorax and serohemorrhagic fluid accumulation with fibrin adhered to the intestinal serosa were observed. The liver presented multiple firm, whitish-yellow, well-demarcated abscesses (0.3–1.2 cm) containing purulent material. The brain exhibited diffuse hyperemia of the leptomeninges and bilaterally engorged meningeal vessels. The kidneys exhibited normal morphology and coloration, and the lungs were pink and showed no visible consolidation, with findings of physiological atelectasis (Figures 1C–G).

Culture of the closed hepatic abscess content yielded pure growth of nonhemolytic colonies on blood agar and small, smooth, translucent, slightly pink colonies on MacConkey agar, consistent with lactose-nonfermenting Gram-negative bacilli. Biochemical characterization revealed an alkaline/alkaline (K/K) reaction in triple sugar iron agar with no gas or H_2_S production, positive citrate utilization, negative lysine decarboxylation, negative urease activity, negative phenylalanine deaminase, negative indole and motility in SIM medium, positive methyl red, and negative Voges–Proskauer reaction. These results were consistent with the characteristics of *Acinetobacter* spp. (Whitman et al., 2012)**.** Culture of the mammary exudate revealed dry, grayish colonies of Gram-positive bacilli compatible with *Corynebacterium pseudotuberculosis* (El Damaty et al., 2023).

**Figure 2:**
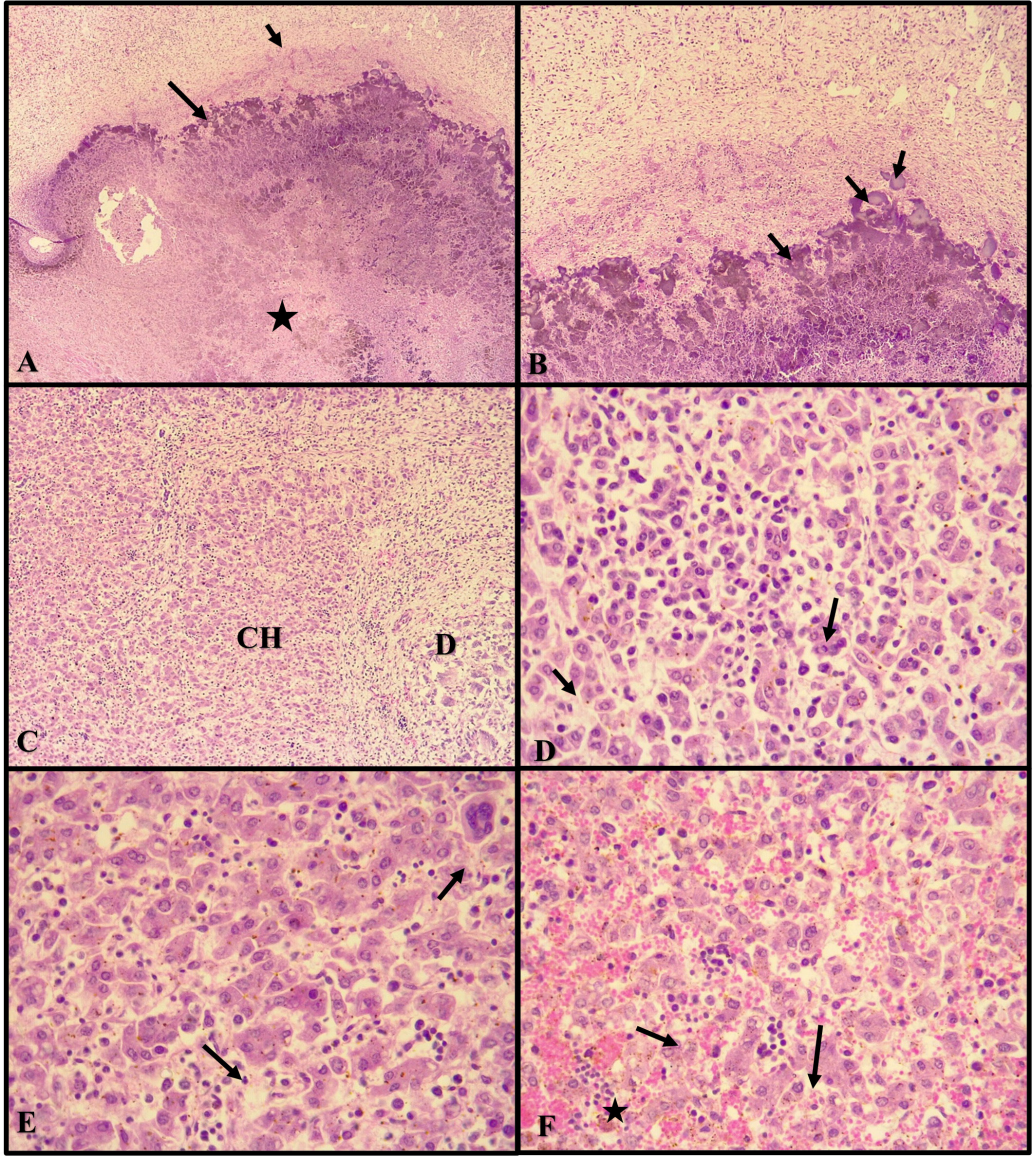
Histopathological features of hepatic parenchyma from an ovine fetus (case 1) with an abscess and *Acinetobacter* spp. isolation. (A) Abscess with peripheral calcification (long arrow) and central necrotic area (asterisk), delimited by a capsule with marked fibroplasia (arrow). (B) Detail showing intense peripheral calcification associated with bacterial colonies (arrow) surrounded by inflammatory cells and enclosed by a thick capsule. (C) Hepatic parenchyma with disorganization of the hepatocyte cords associated with interstitial fibroplasia and infiltration of hematopoietic lineage cells. The inset shows disorganized proliferation of hepatic duct cells surrounded by marked fibroplasia. (D) Hepatocytes appear disaggregated (arrow) or arranged in irregular clusters (long arrow), with interstitial hematopoietic cells present. (E) Hepatocytes are irregularly clustered (long arrow), with sinusoidal capillaries containing hematopoietic cells. The inset shows a megakaryocyte (arrow). (F) Hepatocytes are arranged in irregular clusters (long arrow), with interstitial and sinusoidal spaces containing hematopoietic cells (arrow). Congestion of the hepatic sinusoids (asterisk) is associated with intrahepatic hemosiderosis.

**Figure 3:**
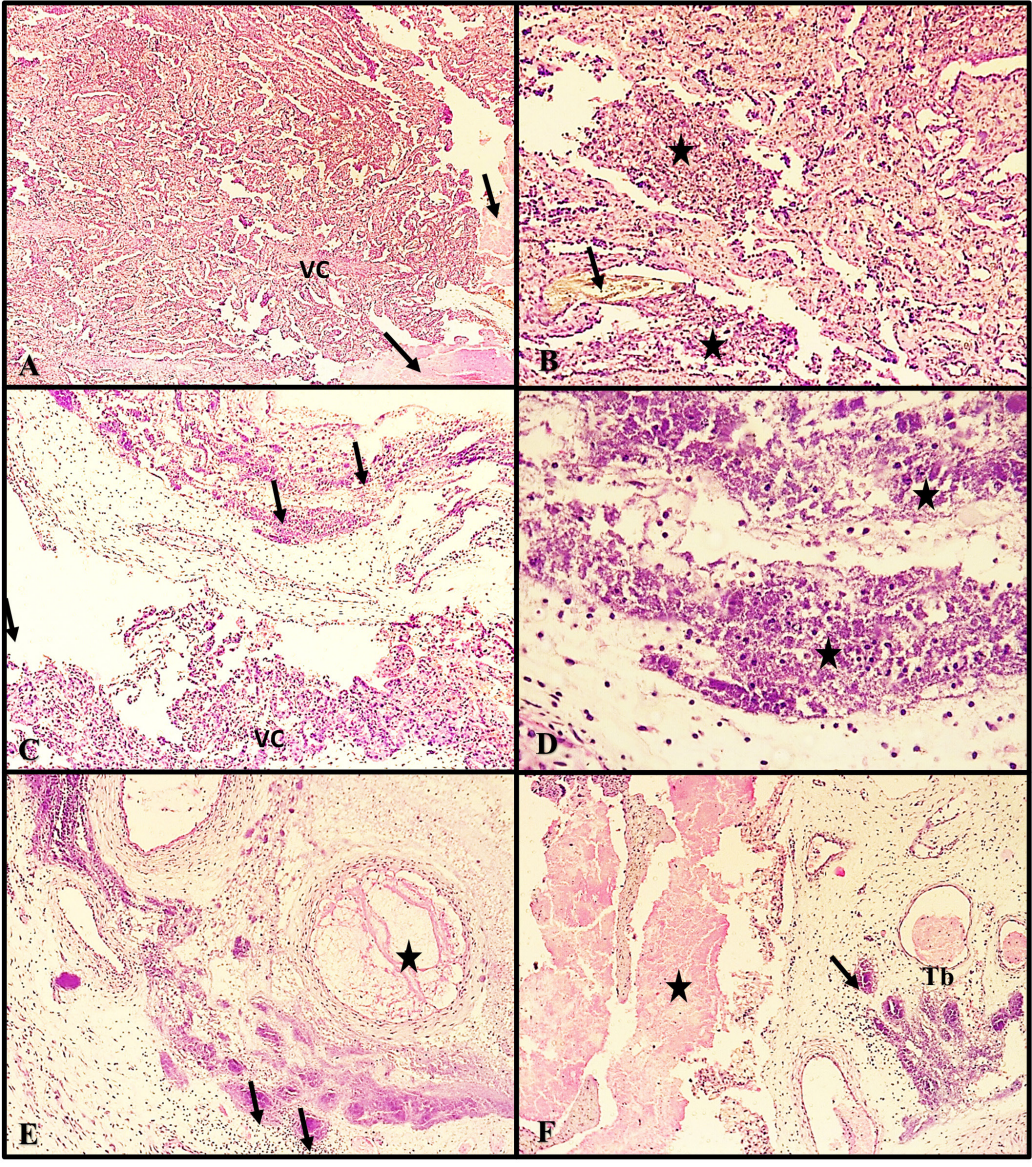
Histopathology of sheep placenta from an abortion case with *Acinetobacter* spp. isolation. Cotyledons. (A) At higher magnification, note the cotyledonary structure of the placenta with chorionic villi and a hemorrhagic area (arrow). (B) Detail showing necrotic areas (asterisk) and calcification (arrow) within the villi. (C) Chorionic villi and chorionic plate. A diffuse area of bacterial colonies (asterisk) was observed in the chorionic plate. (D) Detail of bacterial colonies (asterisk). (E) Multifocal areas of bacterial colonies (arrow) in the chorionic plate, surrounded by mesenchymal tissue and mild inflammatory cell infiltrate. The inset shows an artery with luminal narrowing caused by fibrin deposition (asterisk). (F) Hemorrhage between the chorionic villi (asterisk), presence of bacterial colonies, and thrombus (Tb) formation in the vessels of the chorionic plate.

Histopathological examination of the liver revealed an abscess encapsulated by fibrous tissue, and the presence of peripheral calcification, central necrosis, and intralesional bacterial colonies morphologically were compatible with Gram-negative bacilli and a marked neutrophilic infiltrate. Interstitial fibroplasia, disorganization of the hepatic cords, bile duct proliferation, and sinusoidal congestion with hemosiderosis were also observed (Figure 2). The placenta exhibited hemorrhagic, necrotic, and calcified areas in the chorionic villi, with multifocal bacterial colonies, fibrin deposition, thrombosis, and hemorrhage (Figure 3).

**Case 2:** In October 2022, *Acinetobacter baumannii* and *Proteus mirabilis* were isolated from three late-gestation aborted fetuses from clinically asymptomatic ewes in a flock of 160 Santa Inês sheep raised under a semi-intensive management system in the municipality of Frei Paulo, Sergipe, Brazil (10°32′56′′ S, 37°32′02′′ W). The flock had a recent history of 14 abortions or stillbirths. The farm practiced controlled mating, and all ewes with an expected lambing date within the subsequent 15 days were gathered in a maternity paddock.

**Figure 4:**
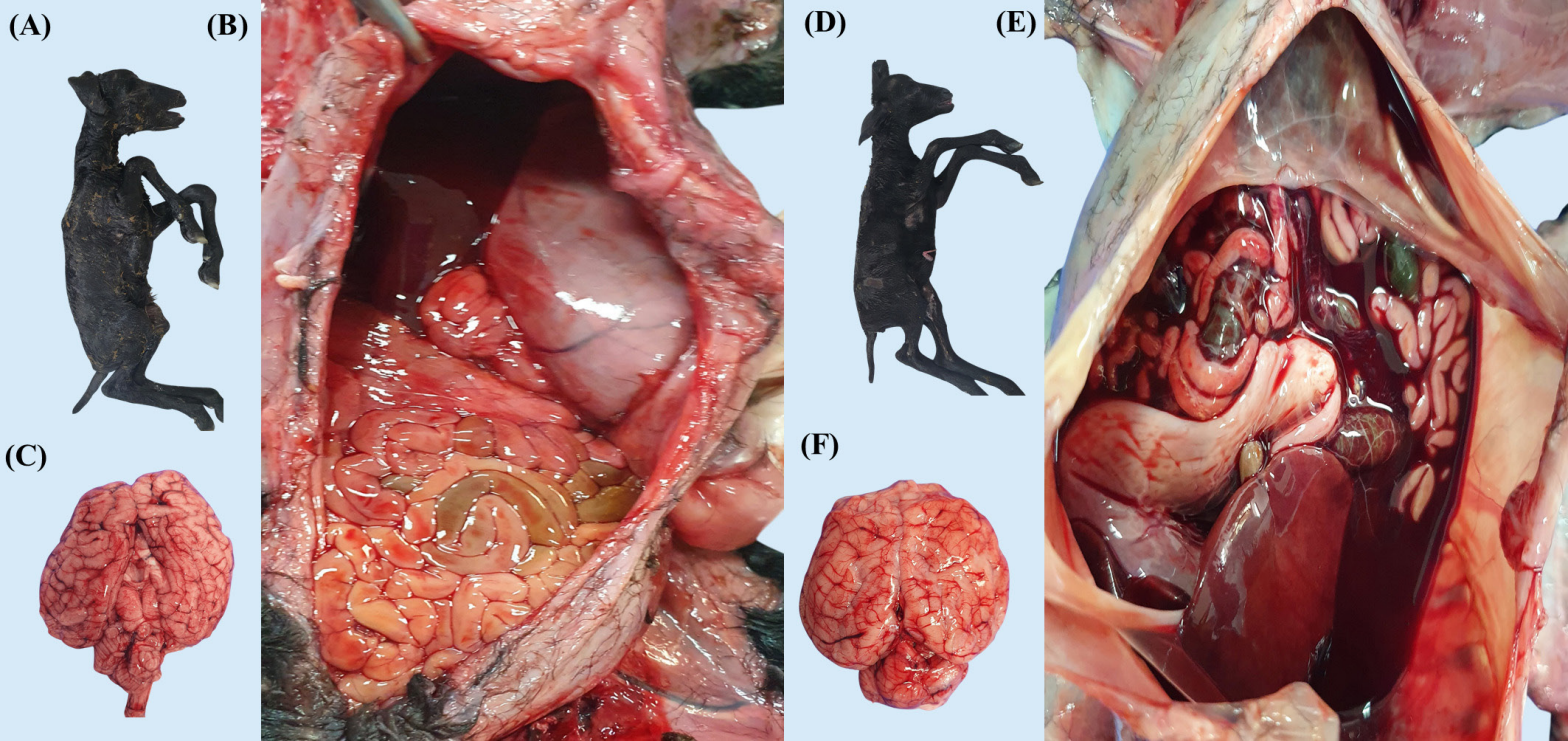
Aborted Santa Inês ovine fetuses from an outbreak in a flock in Sergipe State, Brazil, with isolation of *Acinetobacter baumannii* (A–C) and *Proteus mirabilis* (D–F) from brain swabs. (A, D) General view of the aborted fetuses at late gestation, showing development consistent with full-term animals. (B, E) Thoracic and abdominal cavities with accumulation of serosanguinolent fluid and congested organs. (C, F) Brain with diffuse leptomeningeal hyperemia and marked engorgement of superficial vessels.

During this period, three abortions were recorded on a single day. The fetuses were collected, placed in an insulated container under refrigeration, and sent directly to the animal pathology laboratory at Federal University of Sergipe (UFS), São Cristóvão, Sergipe, Brazil, for necropsy**.** On necropsy examination, the fetuses exhibited diffuse meningeal congestion, marked congestion of the myocardium, liver, and kidneys, and congested lungs with areas of hepatization. A large volume of hemorrhagic fluid was present in the abdominal cavity, consistent with acute septicemia or systemic vasculitis (Figure 4).

Immediately after opening the cavities during necropsy, sterile swabs with Stuart transport medium were used to collect biological material from the brain (two fetuses) and lung parenchyma (one fetus). The samples were kept refrigerated (4°C) in an insulated container and immediately sent to the microbiology laboratory Animal Pat Lab, Aracaju, Sergipe, Brazil, for microbiological processing. In one fetus, brain culture yielded nonhemolytic, lactose-negative colonies, which were biochemically identified using the Bactray^®^ I and II system (Laborclin, Brazil), confirming *A. baumannii* (Whitman et al., 2012). This isolate was resistant to amoxicillin–clavulanic acid and cephalexin and intermediately resistant to ampicillin, ofloxacin, norfloxacin, cephalothin, and doxycycline. In the other two fetuses, brain and lung swabs yielded motile colonies with a swarming growth pattern on blood and MacConkey agar, biochemically identified as *P. mirabilis* (Whitman et al., 2012). This isolate was resistant to ampicillin, ampicillin–sulbactam, cephalexin, cephalothin, and doxycycline. The results of all bacteriological cultures and antimicrobial susceptibility tests are summarized in Table 1.

**Table 1:** Samples, bacterial isolates, identification methods, and antimicrobial susceptibility profiles from three aborted Santa Inês fetuses from a flock in Sergipe, Brazil (case 2).

**Fetus**	**Sample**	**Bacterial isolate**	**Identification**	**Antimicrobial susceptibility**
1	Brain	*Acinetobacter baumannii*	Bactray^®^ I and II systems	**R**: AMC and CX
				**I:** AMP, OFX, NFX, CFL, and DOX
				**S:** AMK, GEN, LVX, CIP, CRO, and CFT
2	Brain	*Proteus mirabilis*	Culture + biochemical tests	**R**: AMP, AMS, CX, CFL, and DOX
				**S:** AMK, GEN, LVX, CIP, CRO, CFT, and AMC
3	Lung			

AMC = amoxicillin–clavulanic acid; AMP = ampicillin; AMS = ampicillin–sulbactam; CFL = cephalothin; CX = cephalexin; DOX = doxycycline; I = intermediate; OFX = ofloxacin; NFX = norfloxacin; R = resistant; S = sensitive.

## Discussion

Despite the growing relevance of *Acinetobacter* spp. in human medicine, knowledge regarding its occurrence and effect in animals remains limited, particularly concerning its role in ruminant abortions. Existing reports mostly isolated cases or incidental findings, making it complicated to determine its significance as a primary pathogen (Müller et al., 2014; Wolf-Jäckel et al., 2020; Baran et al., 2015; Zappa et al., 2017; Mellace et al., 2024). In the above-described cases, the diversity of lesions did not allow for the identification of a consistent pattern associated with *Acinetobacter* spp. However, meningeal congestion was observed in both late-gestation fetuses. In previous studies in cattle and horses, isolation may occur in the absence of significant lesions (Arserim et al., 2020; Akter et al., 2021); however, it has also been reported in buffaloes presenting with signs of acute septicemia (Das & Paranjape, 1986).

In case 1, mastitis caused by *Corynebacterium pseudotuberculosis* may have compromised maternal immunity, potentially favoring fetal infection and hepatic abscess formation, a presentation consistent with the opportunistic behavior of *Acinetobacter* spp. (Rahman & Baxi, 1985; Diker et al., 1986; Eroksuz et al., 2018). The placental lesions observed, namely, bacterial accumulation, thrombosis, and villous necrosis, suggest possible maternal–fetal involvement in gestational interruption (Concha-Bermejillo & Romano, 2021).

*Proteus mirabilis* is an opportunistic pathogen that can form biofilm and cause severe systemic infections in humans and animals (Chakkour et al., 2024). In small ruminants, reports are limited and generally associated with predisposing conditions such as trauma or immunosuppression (Abdollahi et al., 2022; Najd Ghahremani et al., 2023). In the present report, the isolation of *P. mirabilis* from the brain and lungs of aborted fetuses, accompanied by diffuse meningeal, hepatic, and pulmonary congestion, suggests its involvement as an opportunistic agent in case 2. The simultaneous presence of *A. baumannii* likely exacerbated the septic condition, resulting in lesions compatible with acute septicemia, similar to those described in calves (Botes, 1964) and buffaloes (Das & Paranjape, 1986). The antimicrobial resistance of both isolates reinforces concerns about therapeutic management and the spread of resistance genes in ruminant production systems.

In both cases, microbiological characterization was based on culture isolation and biochemical identification, without molecular confirmation of the isolates because of sample disposal. Additionally, no laboratory testing was performed to exclude classical abortifacients such as *Brucella* spp., *Campylobacter* spp., *C. burnetii*, *C. abortus*, *Listeria* spp., *Salmonella* spp., *T. gondii*, and *N. caninum* (Concha-Bermejillo & Romano, 2021). These diagnostic gaps represent important limitations, as histopathological comparison alone is insufficient to exclude such pathogens conclusively. Nevertheless, reporting these microorganisms isolated in aborted ovine fetuses remains relevant for expanding the etiological spectrum considered in abortion investigations.

Considering the pathogenesis of these infections, possible routes of entry for *A. baumannii* and *P. mirabilis* include hematogenous dissemination from maternal infections, such as mastitis, or ascending contamination through the reproductive tract (Rahman & Baxi, 1985; Concha-Bermejillo & Romano, 2021). The concomitant isolation of these two opportunistic pathogens may indicate maternal immunological compromise during abortion (Abdollahi et al., 2022; Müller et al., 2014). Similar to the pattern observed in human infections, where *A. baumannii* and *P. mirabilis* are often associated with septicemia in patients with an immunocompromised status, the findings of this study support their role as secondary pathogens contributing to fetal septicemia (Askari et al., 2019; Chakkour et al., 2024). Comparable lesions consistent with acute septicemia have previously been described in calves and buffaloes (Botes, 1964; Das & Paranjape, 1986).

In contrast to the Uruguayan study by Dorsch et al. (2022), *Proteus spp.* isolates from ovine abortion cases were classified as contaminants in 43 of the 89 cases investigated, mainly due to the absence of compatible lesions or advanced autolysis, in the present report, P. *mirabilis* was isolated from the brain and lungs of two aborted fetuses that exhibited macroscopic lesions consistent with septicemia, such as diffuse leptomeningeal, pulmonary congestion, and accumulation of serosanguinolent abdominal fluid, thereby supporting its potential pathogenic role.

The isolation of *Acinetobacter* spp. and *P. mirabilis* in sheep underscores their potential role as environmental reservoirs and resistant strain sources, with the risk of dissemination at the animal–human–environment interface (Silva et al., 2011; Askari et al., 2019; Elbehiry et al., 2021; Zappa et al., 2017). The occurrence of these agents in flocks, combined with limited field diagnostic capacity, highlights the need to include opportunistic microorganisms in surveillance programs and adopt biosecurity measures to mitigate both animal health and zoonotic risks.

## Conclusion

To the best of our knowledge, this is the first global report of the isolation of *Acinetobacter* spp. and *Proteus mirabilis* from aborted ovine fetuses, documented through microbiological and histopathological characterization. Despite the diagnostic limitations of the molecular techniques employed, the findings reinforce the potential opportunistic role of these agents and the importance of including them in the differential diagnosis of abortions. The detection of antimicrobial resistance highlights the need for control and biosecurity strategies integrated into the “One Health” approach.
